# Advanced Tissue Imprinting With Pneumatic Press for Mass Spectrometry Imaging of Plant Tissues

**DOI:** 10.1002/jms.70007

**Published:** 2025-12-26

**Authors:** Pubudu Nuwan Perera Hapuarachchige, Vy T. Tat, Young Jin Lee

**Affiliations:** ^1^ Department of Chemistry Iowa State University Ames Iowa USA

## Abstract

Sample preparation is an important first step to obtain high quality mass spectrometry imaging (MSI) data. Preparing plant tissues is especially challenging for MSI of thin tissues along the lateral dimensions. The unique challenges involved with plant tissues, such as fragile cell walls, hydrophobic barriers, and specific tissue structures, often lead to inefficiency and difficulties in sample preparation. Imprinting plant tissues onto porous polytetrafluoroethylene (pPTFE) sheet has been widely used to extract internal metabolites in leaves and petals while keeping spatial resolution for MSI. However, pressure applications were typically made manually using a vise or pliers leading to low reproducibility and resolution in MS images. In this study, we introduce a home‐built pneumatic press (PNP) that has been designed to precisely control the pressure application parameters during imprinting. To evaluate the performance of the new device, *Lemna minor* fronds, *Arabidopsis thaliana*, and *Bacopa monnieri* leaves were imprinted onto the pPTFE with PNP, vise, or pliers, and matrix‐assisted laser desorption/ionization (MALDI) MSI was obtained on the imprints. The PNP showed dramatic improvements in reproducibility and image quality compared to manual pressure application tools.

## 1 Introduction

Mass spectrometry imaging (MSI) has been adopted in multiple disciplines of biology, including biomedical science, microbiology, and plant biology [1, 2, 3]. It is an effective tool in spatial metabolomics, enabling the spatially resolved analysis of metabolites within tissues. However, its application to plant spatial metabolomics remains limited [4, 5, 6]. One of the main reasons is the difficulty in sample preparation, which often leads to low reproducibility and low spatial resolution. Unlike animal tissues with high lipid content, plant tissues contain more water with rigid cell walls made of cellulose, hemicellulose, and lignin and are difficult to keep structural integrity during sample preparation [5]. Flat and thin tissues like leaves and petals are often delicate and prone to curling, folding, or tearing during sample preparation, especially when obtaining MSI parallel to the surface. In addition to these structural challenges, plant tissue surfaces are protected by complex hydrophobic barriers, including epicuticular waxes and cuticles [7]. These structural barriers can substantially hinder laser or solvent penetration and analyte extraction, reducing efficient desorption and ionization for direct MSI of internal metabolites.

Despite some shortcomings, a few sample preparation techniques have been successfully utilized in MSI of plant tissues, including dissolution of lipophilic barriers, surface scratching, cryosectioning, tissue fracturing, and imprinting [7, 8]. They involve physically removing or chemically dissolving the protective barriers on the plant surface. For instance, pretreatments using organic solvents such as chloroform can remove the wax layer, while mechanical scraping of the cuticle can also be effective [9, 10]. These methods expose internal endogenous compounds to the surface, accessible for MSI. However, such approaches often lead to delocalization or partial loss of metabolites due to excessive solvent exposure or uneven removal of the protective layer. To overcome these limitations, laser ablation electrospray ionization (LAESI) has been successfully employed to avoid such sample preparation steps [7, 11]. LAESI is a matrix‐free technique where mid‐IR laser energy absorbed by endogenous tissue water causes the explosive ablation of a microvolume of tissue and the release of internal metabolites. This advantage, however, is highly dependent on tissue water content, and the method typically provides a lower spatial resolution in MSI. Other popular MSI techniques, such as matrix‐assisted laser desorption/ionization (MALDI) and nanospray desorption electrospray ionization (nanoDESI), provide high spatial resolution MSI down to single cell resolution, but sampling depth is limited to the tissue surface.

Cryosectioning is a common sample preparation technique to obtain MSI in deeper layers that is widely used in MALDI and DESI‐MSI. However, unlike cross‐sectioning in a perpendicular direction, paradermal sectioning of thin plant tissues is extremely challenging due to their irregular morphologies and structural properties. To address this issue, Hu et al. introduced an electromagnetic field‐assisted frozen tissue planarization (EMFAFTP) to flatten and regularize plant samples prior to obtaining paradermal sections for MSI [12]. This approach minimizes topography‐related artifacts and facilitates the process of cryosectioning along the flat surface. It has been successfully applied to different types of plant tissues, including leaves, petals, and roots. However, this method requires specialized electromagnetic equipment with precise manipulation of freezing conditions and is not widely available yet.

**FIGURE 1:**
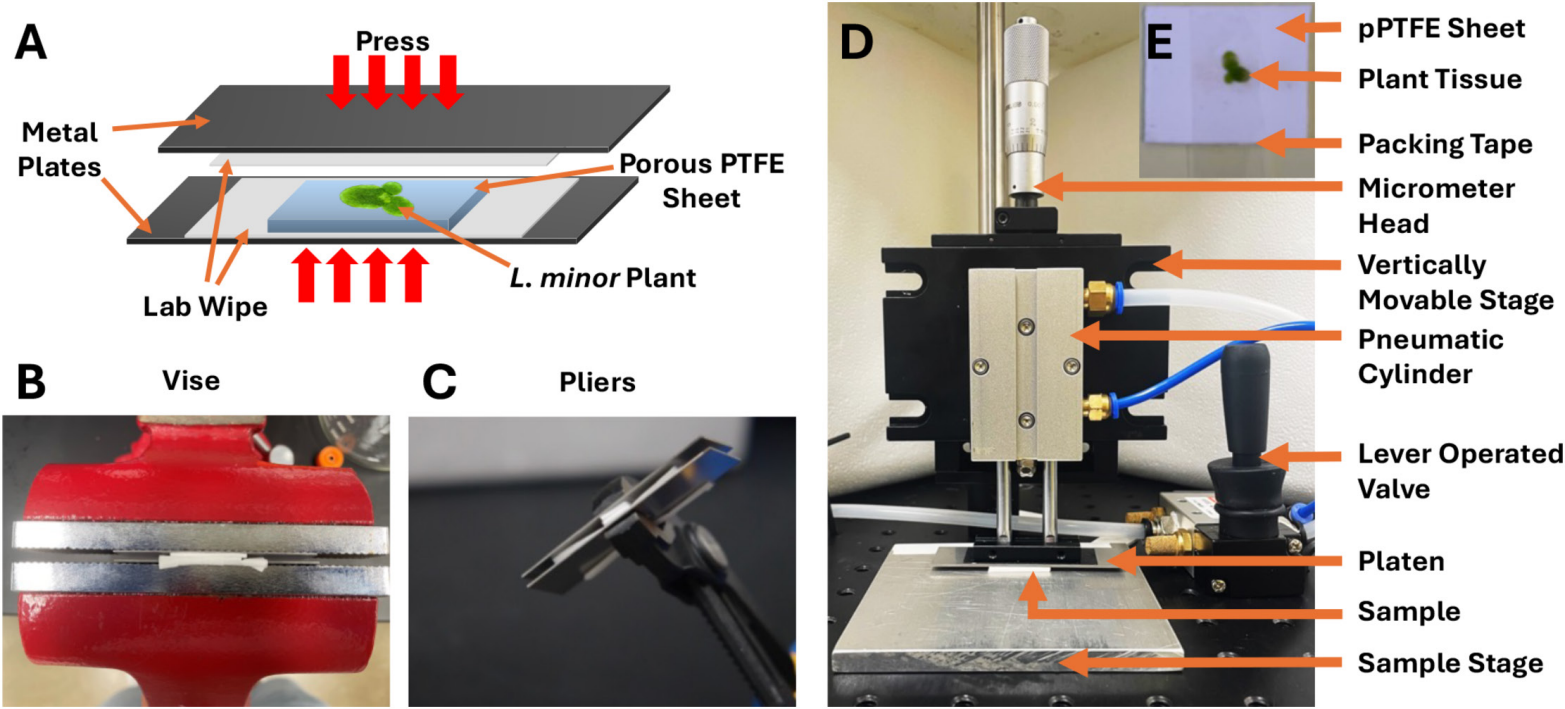
(A) The overall schematic diagram for the plant tissue imprinting using the manual pressing methods, (B) vise or (C) plier, or (D) home‐built pneumatic press in the current work. (E) The photo of tissue sample secured to the pPTFE sheet with packing tape.

Other than cryosectioning with EMFAFTP, fracturing and imprinting are simple and effective sample preparation techniques for visualizing internal plant metabolite distributions parallel to the tissue surface [13]. While fracturing exposes the internal layer of plant tissues for lipid imaging, imprinting transfers mostly hydrophilic small molecules to the porous surface within plant tissues. Imprinting is achieved by placing a plant tissue on a porous polytetrafluoroethylene (pPTFE) sheet and by applying moderate pressure [8], most commonly with a vise or pliers (Figure 1A–C). The first demonstration of this technique was made by Thunig and co‐workers in application to the secondary plant metabolites in *Hypericum perforatum* L. leaves and petals, *Datura stramonium* L. leaves, and *Papaver somniferum* L. capsules [8]. Müller and coworkers studied the degradation products of chlorophyll in senescent plant tissues using the pPTFE imprinted samples, achieving increased signal intensities compared to direct leaf analysis [14]. Recently, Lorenson et al. applied the imprinting technique to *Cannabis sativa* L. fan leaf to confirm the localizations of cannabinoids in trichomes [15]. Most of these applications were made by DESI‐MSI, but we also have demonstrated its application to chemical interactions between soybean and aphids by MALDI‐MSI [13].

The traditional protocols, however, often result in low resolution due to sample delocalization and smearing during the pressure application step [16]. This may not be a critical issue for low‐resolution imaging, including most DESI‐MSI [8, 14, 15, 17, 18, 19, 20, 21, 22], but it becomes a significant challenge in achieving high resolution with MALDI‐MSI [13, 16]. Furthermore, reproducibility is often compromised, mainly due to the inconsistent and extensive manual pressure application. As a result, the pPTFE imprinting technique has not been systematically evaluated for its effectiveness in both qualitative and quantitative research. To address these challenges, we developed an imprinting device using a pneumatic press (PNP). With the precise control of the pressure and distance, the new imprinting method produces much less delocalization and high reproducibility in MSI of leaf tissues when compared with manual pressure application tools.

## 2 Experimental Section

### 2.1 Chemicals, Reagents, and Materials

HPLC grade methanol was purchased from Fisher Scientific (Pittsburgh, PA, USA) and water from Honeywell (Muskegon, MI, USA). N‐(1‐naphthyl) ethylenediamine dihydrochloride (NEDC) from Tokyo Chemical Industry (Portland, OR, USA) or 2,5‐dihydroxybenzoic acid (DHB) from Thermo Fisher Scientific (Ward Hill, MA, USA) was used as the MALDI matrix. 4‐Hydroxy‐3‐methoxycinnamaldehyde (Coniferyl aldehyde, CA) from Sigma‐Aldrich (St. Louis, MO, USA) was used as a derivatization agent for primary amine. Double‐rod, double‐acting aluminum pneumatic air cylinder, TN 10–25, and 5 Way 2 Position Hand Pull Valve were purchased from TAILONZ Pneumatic (Amazon store). The 10‐ to 20‐μm porous polytetrafluoroethylene (pPTFE) sheets (thickness 1.5 mm) were purchased from Scientific Commodities Inc. (Lake Havasu City, AZ, USA).

### 2.2 PNP Design

Figure 1D shows the schematic diagram of PNP developed for the new tissue imprinting method. It is equipped with a twin‐rod pneumatic cylinder (Tailonz, TN 10–25; Wenzhou, Zhejiang, China) that has a diameter of 10 mm bore, a stroke length of 25 mm, and a flat base plate. The working pressure of the pneumatic cylinder is 14–145 psi, which can generate a force between 15 and 110 N on the sample surface. The pneumatic cylinder is mounted to a vertically moving stage. The height of this stage is adjusted using a micrometer head with precision up to 25 μm. This allows for precise control over the degree of tissue compression into the pPTFE sheets, a critical parameter when processing various tissue types. An aluminum plate (25 mm × 75 mm) is attached to the base of the pneumatic cylinder and acts as a platen to apply pressure to the sample surface. The sample stage is set up parallel to the platen to ensure even pressure distribution on the pPTFE sample. A lever‐controlled valve is used to operate the pneumatic cylinder.

### 2.3 Plant Materials

In this study, *Lemna minor, Arabidopsis thaliana*, and *Bacopa monnieri* plants were selected to test imprinting techniques. The *L. minor* plants were grown in nutrient media consisting of 0.5× Schenk and Hildebrandt's nutrient solution (PhytoTech; Lenexa KS, USA) [23]. Illumination was provided by a cool white LED, maintaining a light/dark cycle of 16 h of light followed by 8 h of darkness, with a photon flux density of 60 μmolm^−2^ s^−1^. The temperature was maintained at approximately 20°C–22°C, and the ambient humidity fluctuated between 55% and 70%. Whole plants with mother and two daughter fronds were used for MSI resolution comparison. For the reproducibility experiment, only the mother frond was used, after carefully removing daughter fronds before imprinting, and the entire tissue region was selected as the region of interest (ROI).

The wild‐type *A. thaliana* Col‐0 seeds were germinated and grown in a hydroponic system adapting previous work by Na and Lee [24]. Briefly, after surface sterilization and 3 days of stratification, the seeds were sown on 1% agar‐filled 100‐μL fast PCR tubes that were precut at the bottom. Then, agar holders were placed on a 200‐μL pipette tips holder in a container with 0.5× Hoagland plant nutrient medium (PhytoTech) filled up to the level of the pipette tips holder. The container was placed inside a seedling starter tray with ~150 mL water at the bottom of the tray. Then the seedling starter tray was placed in the growing chamber with humidity at 60%, temperature at 22°C–25°C and long day (16 h light/8 h dark) light condition. The vegetative stage *A. thaliana* leaf tissues were harvested on the 15th day after sowing.

*B. monnieri* plants were purchased from an Amazon seller, Planterest. After arrival, the plants were transferred to 0.5× Hoagland plant nutrient medium and placed in the growing chamber under the same conditions as *A. thaliana* plants for 1 day before harvesting for sample preparation.

### 2.4 Plant Tissue Imprinting

The whole plants with one mother frond and two daughter fronds were used for MS imaging, but only the mother fronds were used for the reproducibility experiment due to the thickness difference between mother and daughter fronds. The abaxial side of the *L. minor* plant was faced on a pPTFE sheet and tightly secured by covering with packing tape, as shown in Figure 1E. The use of tape was not previously adopted, but it has multiple benefits, including straightening naturally folded plant tissues, avoiding sample movement during pressing, and minimizing liquid extraction from the top surface. There are two imprinting parameters, working pressure and pressing depth, that need to be optimized to achieve high‐quality, reproducible MS images with the PNP. The procedure is described in the Supporting Information and Figure S1 using *L. minor* fronds as an example. For *L. minor* tissues, optimal pressing parameters were 50 μm for the final pressing depth and 60 psi for the working pressure. The two common pressure application tools, vise (Figure 1B) and pliers (Figure 1C), were also used for comparison as described in Thunig et al. [8] and Xia et al. [21], except pressing time was maintained for 10 s for both. The same imprinting procedure and pressing parameters were successfully used for the *A. thaliana* leaf samples with the PNP and vise. For the *B. monnieri* leaf samples, first, the PNP pressing parameters optimized for *L. minor* tissues were used. Then, the PNP pressing parameters were re‐optimized for *B. monnieri* leaves, which revealed a working pressure of 120 psi and a final pressing depth of 500 μm.

### 2.5 Optical Image Analysis

To assess the final resolution of the imprinted tissue samples using each pressure application method, optical images were taken using an Olympus microscope and a GoPro HERO8 still camera.

### 2.6 MALDI‐MS Imaging and Data Analysis

The *L. minor* imprinted samples were vacuum‐dried for 1 min and subsequently coated with a 7 mg/mL NEDC solution in 70% methanol using a TM sprayer (HTX Technology; Chapel Hill, NC, USA) for MALDI matrix deposition. Platinum sputtering for 10 s using a sputter coater provided a conductive sample surface for the MALDI‐MS analysis. MALDI‐MSI data acquisition was performed in negative ion mode with a MALDI source [25] (Spectroglyph; Kenniwick, WA, USA) attached to a QExactive HF Orbitrap mass spectrometer (Thermo; San Jose, CA, USA), with a mass resolution of 120 000 at *m/z* 200 and for the *m/z* range of 67–1000. MSI data collection utilized a 349‐nm Nd:YLF laser with a spot size of 20 μm. A 30‐μm raster step and a 50‐μm raster step were used in resolution and reproducibility experiments, respectively.

The *A. thaliana* leaf imprinted samples were vacuum‐dried for 1 min and subsequently coated with a 20 mg/mL CA in methanol to derivatize amine‐containing small metabolites followed by matrix deposition of 40 mg/mL DHB solution in 70% methanol using the TM sprayer. Ten seconds of gold sputtering was used to provide a conductive sample surface for the MALDI analysis. MALDI‐MSI data acquisition was performed in positive ion mode with a mass resolution of 120 000 at *m/z* 200 for the *m/z* range of 67–460, using a 60‐μm raster step size.

The *B. monnieri* leaf imprinted samples were vacuum‐dried for 1 min and subsequently coated with a 40 mg/mL DHB solution in 70% methanol using the TM sprayer for MALDI matrix deposition. Ten seconds of gold sputtering was used to provide a conductive sample surface for the MALDI analysis. MALDI‐MSI data acquisition was performed in positive ion mode, with a mass resolution of 120 000 at m/z 200, over the *m/z* range of 100–1000, using a 60‐μm raster step size.

Data were processed using Xcalibur (Thermo), Image Insight (Spectroglyph), and MSiReader [26]. Metabolite annotations are made by uploading .imzML files to METASPACE (http://metaspace2020.org) [27] and searching against the metabolite database of KEGG or plant_coconut. It is based on molecular formulas, and all annotations should be considered as tentative arbitrarily chosen among the matching metabolites with the same formulas.

Multivariate analysis was performed using MetaboAnalyst [28]. The list of metabolite features was first filtered using a standard‐deviation‐based method to remove low‐variance features. The remaining features were normalized to a reference peak (*m/z* 140.050, a matrix‐related peak), followed by log transformation and Pareto scaling. Principal component analysis (PCA) was then performed to evaluate clustering patterns among the three pressing methods.

## 3 Results and Discussion

In the previous imprinting techniques using either vise or pliers, manual force is applied to two metal plates between which plant tissue is faced down the abaxial side to the pPTFE sheet. For the best result, only the necessary amount of force should be applied for a very short duration. However, it is impossible to control these parameters from sample to sample when manually applying the force. This inconsistency significantly impacts the reproducibility of the pPTFE imprinted samples. Laboratory wipes are typically used to absorb excess liquid extract from both sides of the pPTFE sheet, but it is not enough to prevent metabolite delocalization and smearing on the imaging surface. To address this issue, we developed an in‐house PNP system as described in the experimental section (Figure 1D). The new imprinting method allows us to precisely control the amount of force we apply with a gas tank pressure and the maximum depth of impression using a translation stage with micrometer head. These parameters can be optimized for different plant tissues as needed.

With 60 psi of gas pressure used in this study, 65 N of force is applied to the sample surface, considering two pistons with a bore diameter of 10 mm. As the platen is pushing the sample surface, the tissue and the pPTFE will be squeezed, and the resistance force will be building up against the PNP until it reaches 65 N when the PNP stops its movement. It occurs within tens of milliseconds, according to video capture. Even though a significantly low force was employed in the PNP compared to traditional pressing tools, this sudden impact generates a sufficient impulsive force on plant tissue to explode the plant cells and extract metabolites efficiently onto the pPTFE surface. It should be noted that a small pore size, 10–20 μm, is used in this study to attain the highest spatial resolution afforded by MALDI‐MSI.

**FIGURE 2:**
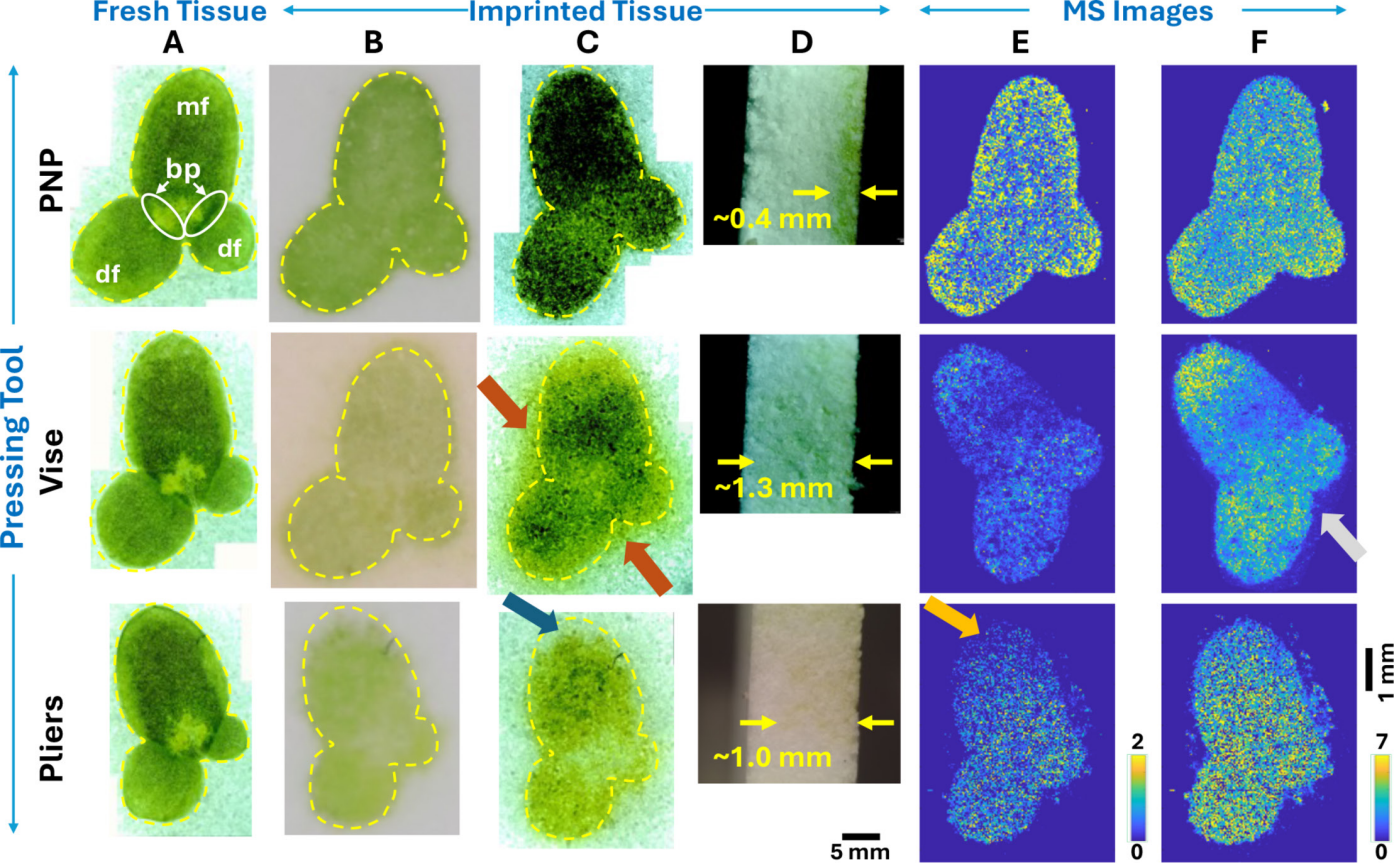
(A–C) The optical images of duckweed samples prepared using three different pressing tools: microscope images of *L. minor* (A) before and (C) after imprinting and (B) camera images after imprinting. The white labels on the PNP used fresh tissue indicate different tissue regions: mf‐ mother frond, df‐ daughter frond and, bp‐ budding pouch. The yellow boundary lines show the area of the original tissue. Red and blue arrows indicate metabolite delocalization beyond the tissue boundary and absence of imprinted materials in some tissue areas, respectively. (D) Cross‐sectional microscope images of the pPTFE sheet showing the penetration depth of extracted metabolites. The yellow arrows show the measurement locations. (E, F) The MALDI‐MS images of two selected metabolites obtained in negative mode: (E) glucose‐6‐phosphate (G6P, *m/z* 259.022) and (F) ungeremine (*m/z* 265.074). Ion signals were normalized to matrix peak at *m/z* 140.050.

The new imprinting method was tested for duckweed ( *L. minor* ) samples and compared with other imprinting using vise and pliers (Figure 2). In the camera images, all three techniques were successfully able to imprint leaf materials onto the top surface of the pPTFE sheets (Figure 2B). At a closer look in the microscope images (Figure 2C), however, significant materials were spread beyond the tissue boundary with vise (e.g., red arrows), indicating heavy lateral delocalization of plant materials, and no or small amounts of materials were transferred in some tissue areas with pliers (e.g., blue arrow). It is attributed to the excessive or uneven force generated by vise and pliers, respectively. In contrast, there was almost no difference in tissue boundary with PNP before and after imprinting, suggesting the PNP generated a minimum necessary and well‐controlled force. In the cross‐sectional image of pPTFE sheets after imprinting (Figure 2D), the penetration depth of metabolite extract into pPTFE was much less (0.4 mm) with the PNP compared to vise and pliers (1.3, 1.0 mm), which is attributed to much shorter pressing time (~milliseconds vs. ~seconds). The darker green color is observed for the PNP imprinted sample compared to the vise and pliers in both the camera and microscope images. It is due to the higher material density near the pPTFE surface originated from the lower penetration depth and minimal lateral delocalization. Further, the thickness of the pPTFE sheet did not change after imprinting with the PNP, but it was reduced by ~40 and ~10 μm, respectively, with vise and pliers due to the permanent deformation. Similar results were obtained in multiple experiments as shown in an example in Figure S2.

Additionally, we conducted a histological evaluation of *L. minor* mother frond before and after the PNP‐imprinting process to investigate the tissue damage by the imprinting process. Figure S3 presents a comparison between the optical images of a fresh frond and the Hematoxylin and Eosin‐stained tissue recovered after the imprinting. The gross morphology of the stained, imprinted tissue matches that of the fresh tissue, confirming that the overall anatomical footprint is preserved without lateral distortion of tissue architecture. High‐magnification imaging of the imprinted tissue reveals some cellular rupture. This cellular collapse was expected to deliver internal cytoplasmic metabolites onto the pPTFE sheet. The correspondence between the intact tissue boundary and minimum cellular damage confirms that the PNP imprinting achieves efficient metabolite extraction while maintaining high lateral spatial accuracy.

MALDI‐MSI was performed for the imprinted pPTFE sheets in negative mode. The MS images of two tentative metabolites, glucose‐6‐phosphate (G6P) and ungeremine, were shown in Figure 2E,F, representing low‐ and high‐abundance metabolites, respectively (see Figure S4 for more metabolites MS images). The annotation is based on accurate mass with METASPACE [27], and structural isomers cannot be differentiated. The MS images highlight apparent differences in tissue boundary, metabolite localization, and signal distribution depending on the pressing tools used for imprinting. The MS images show well‐preserved tissue boundaries with the PNP imprinted tissue sample, confirming minimal metabolite delocalization observed in the optical images. In contrast, samples imprinted using pliers exhibit significant metabolite delocalization beyond the tissue region in MS images and low signals at the top of the mother frond (orange arrow), verifying the uneven force application observed in optical images. Similarly, the imprinted sample with vise shows some degree of delocalization beyond the tissue boundary (gray arrow). MS images of other metabolites show mostly higher signals for the PNP‐imprinted sample compared to the vise and pliers‐imprinted samples (Figure S4). Some low‐abundance metabolites are either undetected or barely detected in the latter methods due to the lower material density near the pPTFE surface, as discussed above. Direct MALDI‐MSI was also conducted on the adaxial surface of the *L. minor* fronds for comparison. The resulting MS images for the selected metabolites revealed little to no signal for these compounds (Figure S4). Some metabolite signals, such as G6P, disaccharide, and disaccharide phosphate, might have come from minor metabolite leaching through the very thin cuticle layer or the stomata, facilitated by the heated methanol‐based matrix application. This outcome highlights the effectiveness of the PNP imprinting technique for MSI of internal metabolites.

Some PNP‐generated metabolite images show interesting localizations. For G6P, a slightly higher abundance is observed near the edge of the tissue (Figure 2E). The middle part of the *L. minor* frond has a special structural tissue filled with air known as the aerenchyma tissue, with relatively large cells that cover an average of 40% of the frond area [29]. This tissue region with large air pockets helps the *L. minor* to float on the water, store oxygen, and facilitate the gas exchange between the adaxial and abaxial sides of the fronds. Therefore, we hypothesize that one of the reasons for this lower abundance observed in the middle of the frond for G6P might be due to the lower density of cells in this aerenchyma tissue region. Adenine and guanine are most abundant at the budding pouch (pink arrow), where daughter fronds develop and grow through vegetative reproduction (Figure S4A,B). These purine bases are essential components of DNA and RNA biosynthesis and play a crucial role in cell division and proliferation during the daughter fronds' development stage [30]. Disaccharide and disaccharide phosphate, most likely sucrose and sucrose‐6‐phosphate (S6P), are mostly localized to the mother frond (Figure S4E,F). Sucrose is a transportable form of sugar, synthesized in cytosol from uridine diphosphate glucose (UDP‐glucose) and fructose‐6‐phosphate (F6P) via S6P as an intermediate. As there is a low carbon demand, mature mother frond may play a role as source tissues and excess carbon may be converted to sucrose and transported to sink tissues, daughter fronds. Similar localization is observed for sucrose prepared by fracturing, a completely different sample preparation where leaf tissue is divided into two halves across the lateral dimension (manuscript in preparation), further supporting the localization of sucrose in Figure S4E. In contrast, blurry, delocalized, or uneven signal distribution is observed in many other MS images when the other two pressing techniques are used (Figure S4).

**FIGURE 3:**
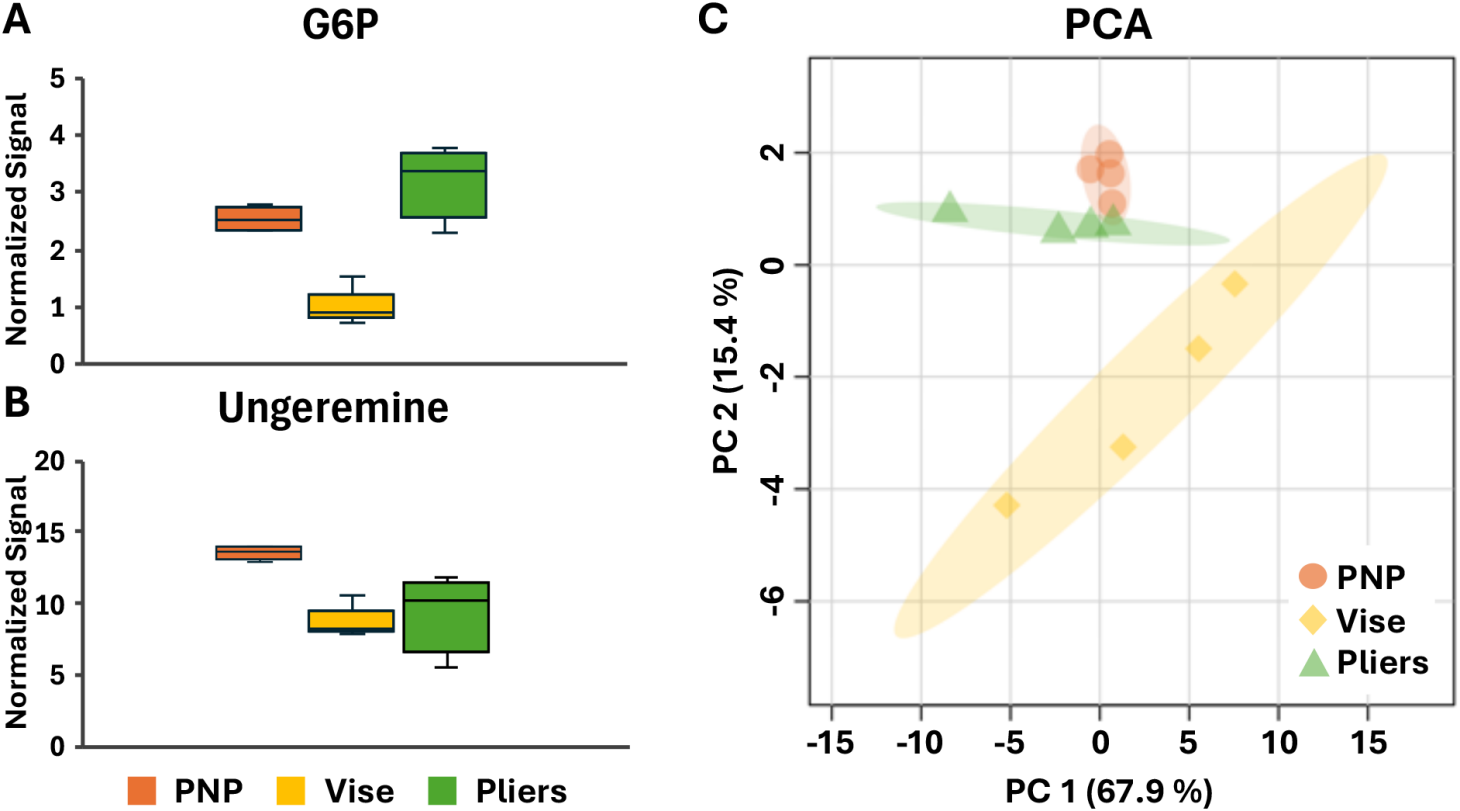
(A, B) Comparison of two selected metabolite signals, (A) G6P and (B) ungeremine, averaged over mother frond tissue region (PNP and pliers: *n* = 4, vise: *n* = 3). Ion signals were normalized to the matrix peak at *m/z* 140.050. (C) Principal component analysis (PCA) analysis of metabolite features extracted from MSI using three different pressing methods (*n* = 4 for all imprinting techniques).

Reproducibility is crucial in any sample preparation, especially for quantitative analysis. The average metabolite ion signals for G6P and ungeremine were extracted from the mother frond tissue region and compared among the imprinting techniques (Figure 3). As expected, a much less standard deviation is observed for PNP compared to vise or pliers, indicating a minimum sample‐to‐sample variation with PNP. We hypothesize that a key factor for high reproducibility is maintaining metabolites near the pPTFE surface (Figure 2D), which also explains high ion signals with PNP in general. The highest variation with pliers might have originated from the inconsistent force and deeper penetration depth (~1.0 mm), which could be highly variable depending on the manual force each time. It also explains the lowest G6P signals with vise, assuming G6P diffuses deeper into the pPTFE sheet as a result of extensive force and its concentration is diluted on the surface where MALDI sampling occurs.

To validate the enhanced coverage of the entire metabolome by the new method, the total number of metabolite features was compared between three imprinting techniques in the MSI of mother fronds. As there is no metabolite database available for duckweeds, we compared the total number of peaks with S/N above 10 as a surrogate for the detected metabolome in each dataset, which will be a slight overcount considering matrix peaks and multiple adduct formations. In the imprinting with PNP, a total of 1303 (±214) peaks were detected compared to 947 (±677) and 1081 (±170) in the vise and plier, respectively, suggesting a much larger number of metabolomes is accessible by PNP.

Multivariate analysis was performed to further evaluate the overall metabolite‐level differences among the three imprinting methods. A total of 56 metabolite features consistently detected across all samples were extracted from four biological replicates of the reproducibility experiment, per imprinting method. PCA was conducted to visualize clustering patterns and assess differences in reproducibility and global metabolomic profiles among the three pressing methods. The first two principal components (PC1 and PC2) explained a combined 83.3% of the total variance, resulting in a distinct clustering of samples based on the imprinting method (Figure 3C). The PNP‐imprinted samples formed a tight and distinct cluster that was clearly separated from the clusters produced by vise‐ and pliers‐imprinted samples. This exceptionally tight cluster of PNP used samples confirms the substantially improved reproducibility and lower sample‐to‐sample variation in overall metabolite profiles. In contrast, the vise and pliers used samples displayed broader dispersion, reflecting greater variability across their replicates. The PCA separation further supports the notion that controlled imprints not only enhance individual metabolite signals but also contribute to more consistent global metabolomic patterns, thereby strengthening the conclusion that the PNP method provides superior reproducibility and overall data quality for MSI‐based plant metabolomics.

**FIGURE 4:**
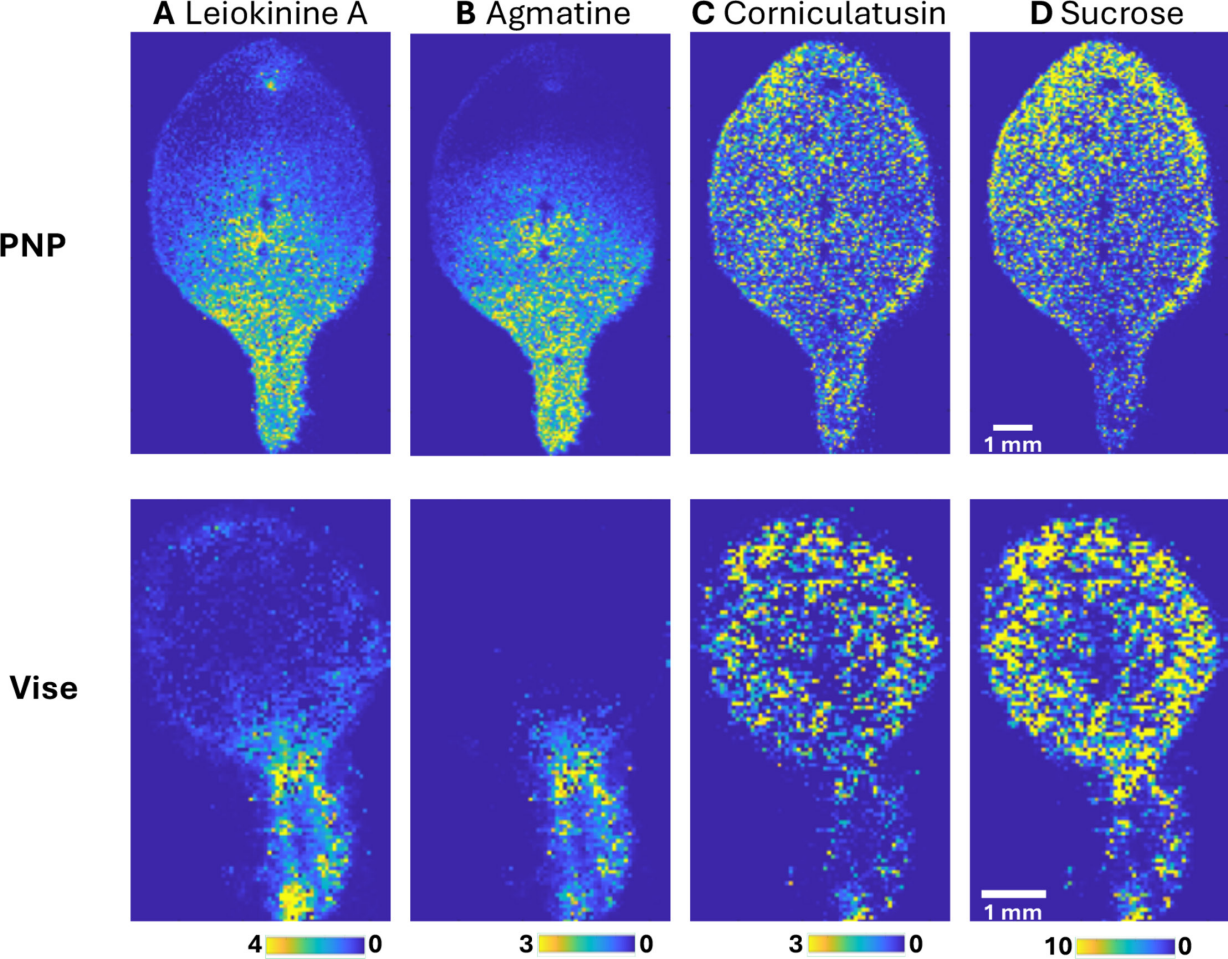
The comparison of the PNP and vise imprinted samples of *A. thaliana* leaf. The MALDI‐MS images of four selected metabolites obtained in positive mode: (A) leiokinine A ([M + H]^+^: *m/z* 232.133), (B) agmatine ([M + CA + H]^+^: *m/z* 291.182), (C) corniculatusin ([M + Na]^+^: *m/z* 355.042), and (D) sucrose ([M + K]^+^: *m/z* 381.079). MS images are normalized to matrix‐related peaks at *m/z* 409.055. Metabolite annotations are tentative, based on accurate mass with METASPACE.

To further validate the newly developed imprinting technique, it is applied to Arabidopsis leaves and compared with the sample preparation with vise (Figure 4). The MS images for four selected metabolites of the PNP imprinted sample show very distinct spatial distributions and sharp boundaries around the leaf. In contrast, the vise‐imprinted sample has a clear delocalization of metabolites beyond the tissue boundary with blurred spatial metabolite details and lower signal intensities. This comparison confirms that the PNP outperforms traditional imprinting tools in preserving the spatial information of metabolite distributions.

**FIGURE 5:**
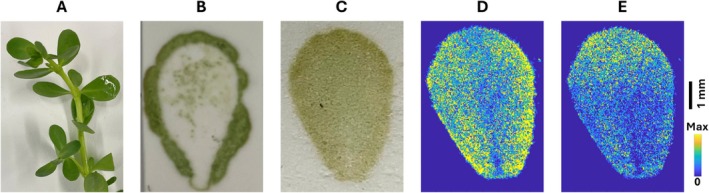
(A) Adult *Bacopa monnieri* plant leaves were used in this study. (B, C) Camera images of imprinted *B. Monnieri* leaf using (B) the previous PNP parameters of 60 psi and 50 μm and (C) the newly optimized parameters of 120 psi and 500 μm. (D, E) The MALDI‐MS images of two selected metabolite features from the tissue area, obtained in positive mode: (D) *m/z* 937.456 ([C_46_H_74_O_17_ + K]^+^), and (E) *m/z* 967.466 ([C_47_H_76_O_18_ + K]^+^).

As a further demonstration of the PNP method's versatility and adaptability for various plant tissues, we performed an imprinting experiment on *B. monnieri* . Compared to the tissues of *L. minor* or *A. thaliana* , the leaves of *B. monnieri* are succulent and rich in wax, making them considerably thicker and higher in water content. The imprinting parameters optimized for *L. minor* did not work for *B. monnieri* due to the different physical properties (Figure 5B). There was almost no imprinting for the middle part of leaves but only some squeezed out along the margin. The imprinting parameters were optimized for *B. monnieri* leaves, which required a deeper pressing with a higher pressure (120 psi and 500 μm) to provide enough internal pressure for cytoplasmic materials to overcome thick wax barriers (Figure 5C). The MS images corresponding to the molecular formulae of two well‐known secondary metabolites of *B. monnieri* , bacopasaponin C and bacoside A3, were successfully visualized matching with the imprint optical image, as shown in Figure 5D,E, respectively.

It should be noted that the imprinting process may introduce certain artifacts, depending on the type of tissue. The efficiency of metabolite transfer can be varied by factors such as the mechanical properties of different tissue regions (e.g., midrib vs. lamina blade) and the physicochemical properties of the metabolites themselves. These tissue‐related irregularities are known challenges in imprinting, but it is often impossible to distinguish them from the large, non‐reproducible errors introduced by manual pressing tools. The advantage of the PNP is that it eliminates this significant variability induced by manual pressing tools. By providing a stable, precisely controllable, and highly reproducible pressing tool in pPTFE imprinting, the PNP establishes the necessary foundation to systematically investigate these tissue irregularity biases. While a comprehensive quantitative evaluation of these factors is beyond the scope of this initial method‐development study, it remains an important direction for future investigation.

## 4 Conclusion

To conclude, the imprinting system built with PNP has significant enhancements in both resolution and reproducibility for MALDI‐MSI compared to the previously used vise and pliers. The ability to detect low abundance metabolites with low sample‐to‐sample signal variation and to keep the tissue heterogeneity with high precision makes the PNP‐based imprinting method a reliable and high‐quality sample preparation technique for plant spatial metabolomics. This design of the PNP allows for precise control over the applied force and pressing distance, making it particularly useful for a wide range of plant tissues. This enhanced performance of the PNP imprinting technique opens the door to more reliable and detailed quantitative analyses, as well as untargeted spatial metabolomics studies, in a wide variety of plant systems.

## Conflicts of Interest

The authors declare no conflicts of interest.

## Supporting information


**Figure S1:** (A) The schematic diagram of home‐built pneumatic press and the procedure to optimize the imprinting parameters. Step 1: Setting the maximum pressing depth position. Step2: Imprinting tissues at several different pressures (e.g., 20, 40, 60, and 80 psi). Step 3: Setting the 0 μm‐pressing depth position at the surface of pPTFE sheet. Step 4: Change the pressing depth position beyond zero‐depth position. Step 5: Imprinting tissues with various depth position with the pressure determined in Step 2. Note the schematic diagram is not in the scale and the thickness for pPTFE sheet is exaggerated on purpose. (B, C) Optical images of imprinted *L. minor* frond at (B) several different pressures (20, 40, 60, and 80 psi) and maximum pressing depth position, and at (C) different pressing depth (0, 25, 50, and 75 μm) at 60‐psi working pressure (pd = penetration depth measured from cross‐sectional images).
**Figure S2:** Example of replicate imprinted sample using the pneumatic press.
**Figure S3:** Validation of spatial fidelity and cellular integrity of *L. minor* mother during the pPTFE imprinting using the PNP. The microscope images of a (A) fresh mother frond and (B) the Hematoxylin and Eosin‐stained tissue recovered after imprinting.
**Figure S4:** Comparison of tissue‐specific localization of selected internal metabolites in *L. minor* using three distinct imprinting techniques alongside direct MSI analysis of the adaxial leaf surface.

## Data Availability

The data that support the findings of this study are openly available in METASPACE (https://metaspace2020.eu/project/pnp_ptfe_imprinting_plant).
