# Supplementary material for: Advanced Tissue Imprinting With Pneumatic Press for Mass Spectrometry Imaging of Plant Tissues
Source: J Mass Spectrom. 2025 Dec 26;61(1):e70007. doi: 10.1002/jms.70007 (PMC12741710; doi:10.1002/jms.70007)
Supplement: Supplementary file 1 — Figure S1: (A) The schematic diagram of home‐built pneumatic press and the procedure to optimize the imprinting parameters. Step 1: Setting the maximum pressing depth position. Step2: Imprinting tissues at several different pressures (e.g., 20, 40, 60, and 80 psi). Step 3: Setting the 0 μm‐pressing depth position at the surface of pPTFE sheet. Step 4: Change the pressing depth position beyond zero‐depth position. Step 5: Imprinting tissues with various depth position with the pressure determined in Step 2. Note the schematic diagram is not in the scale and the thickness for pPTFE sheet is exaggerated on purpose. (B, C) Optical images of imprinted L. minor frond at (B) several different pressures (20, 40, 60, and 80 psi) and maximum pressing depth position, and at (C) different pressing depth (0, 25, 50, and 75 μm) at 60‐psi working pressure (pd = penetration depth measured from cross‐sectional images). Figure S2: Example of replicate imprinted sample using the pneumatic press. Figure S3: Validation of spatial fidelity and cellular integrity of L. minor mother during the pPTFE imprinting using the PNP. The microscope images of a (A) fresh mother frond and (B) the Hematoxylin and Eosin‐stained tissue recovered after imprinting. Figure S4: Comparison of tissue‐specific localization of selected internal metabolites in L. minor using three distinct imprinting techniques alongside direct MSI analysis of the adaxial leaf surface. [file JMS-61-e70007-s001.pdf]

Supporting Information

Advanced Tissue Imprinting with Pneumatic Press for Mass Spectrometry Imaging of Plant Tissues.

Pubudu Nuwan Perera Hapuarachchige, Vy T. Tat, and Young Jin Lee*

Department of Chemistry, Iowa State University, Ames, IA 50011, United States

*Corresponding author: Young Jin Lee. Email: yjlee@iastate.edu

Procedure to Optimize Imprinting Parameters

To achieve high-quality, reproducible MS images with the PNP, two imprinting parameters, working pressure and pressing depth, need to be optimized. Here, we explain the step-by-step procedure with *L. minor* fronds as an example (**Figure S1**). First, the working pressure is determined at the maximum pressing depth position. The vertical stage was lowered using the micrometer until the platen gently contacts with the sample stage **(Step 1)**. *L. minor* fronds were then imprinted onto the pPTFE sheets with various working pressures **(Step 2)**. The optical images of the imprinted sample are carefully inspected to determine the most optimum pressure **(Figure S1B)**. The goal is to identify the lowest pressure that produces a clear and complete imprint with minimum metabolite penetration and metabolite delocalization . We determined 60 psi as the optimal working pressure for *L. minor*.

Second, the optimal pressing depth is determined at the optimized working pressure. The platen is set to gently contact the surface of the pPTFE sheet by fully extending the piston, defining the zero-pressing depth **(Step 3)**. After removing the pPTFE sheet, the vertical stage is lowered even further typically by 25 µm increment at a time **(Step 4)**. A new series of imprints are then generated by systematically increasing the pressing depth **(Step 5)**. The optical images of the imprinted samples are carefully inspected for each penetration depth **(Figure S1C)**. The penetration depth of the plant extract into the pPTFE sheet is also measured by cutting the pPTFE sheet in the middle and expecting the cross-sectional optical images. The optimal pressing depth is identified by selecting the clear and full leaf imprint that was achieved with a minimum penetration depth. The working pressure can be further optimized by fine tuning the gas pressure at the optimum depth; however, we find it typically unnecessary.

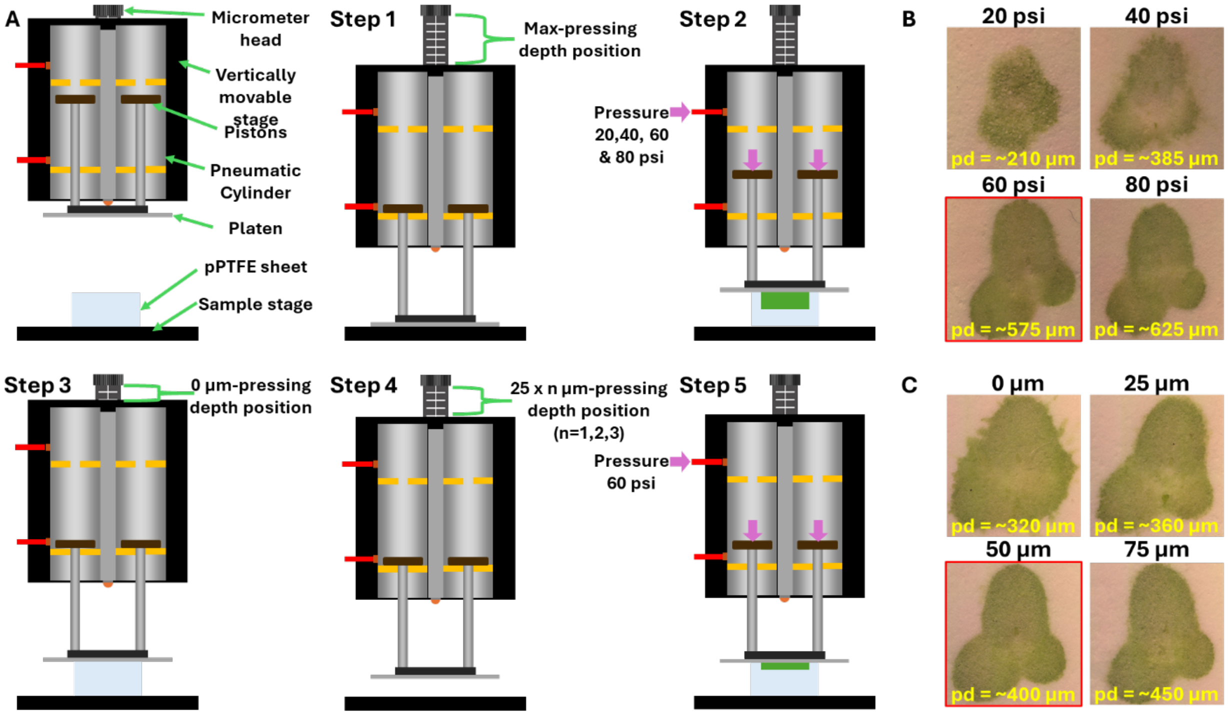

Figure S1. (A) The schematic diagram of home-built pneumatic press and the procedure to optimize the imprinting parameters. **Step 1:** Setting the maximum pressing depth position. **Step2:** Imprinting tissues at several different pressures (e.g., 20, 40, 60 & 80 psi). **Step 3:** Setting the 0 µm-pressing depth position at the surface of pPTFE sheet. **Step 4:** Change the pressing depth position beyond zero-depth position. **Step 5:** Imprinting tissues with various depth position with the pressure determined in Step 2. Note the schematic diagram is not in the scale and the thickness for pPTFE sheet is exaggerated on purpose. **(B, C)** Optical images of imprinted *L. minor* frond at **(B)** several different pressures (20, 40, 60 & 80 psi) and maximum pressing depth position, and at **(C)** different pressing depth (0, 25, 50 & 75 µm) at 60 psi working pressure (pd= penetration depth measured from cross-sectional images).

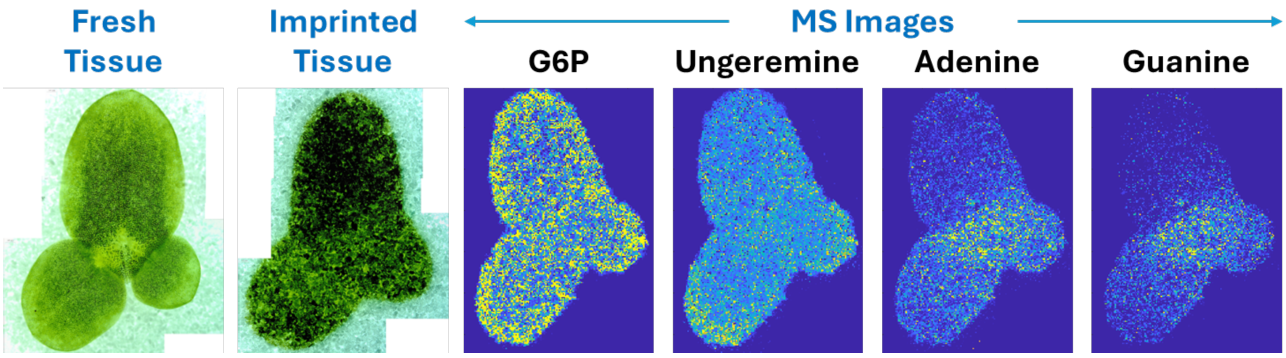

Figure S2. Example of replicate imprinted sample using the pneumatic press.

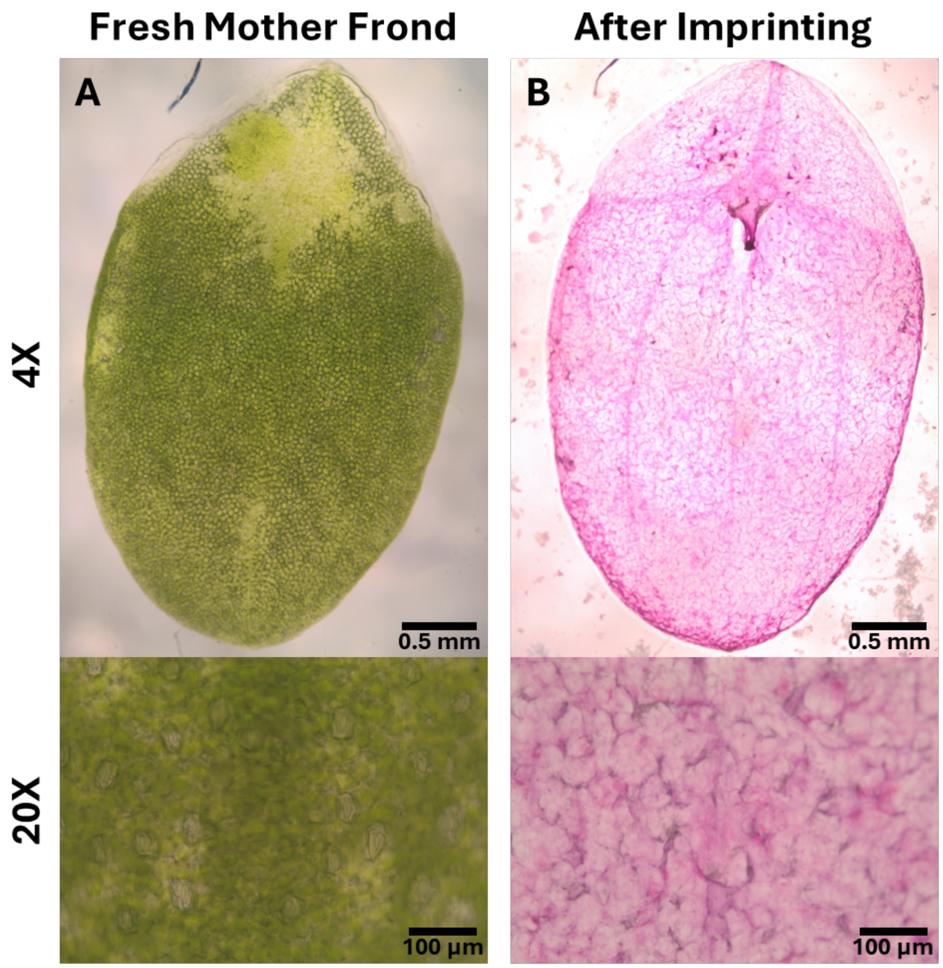

Figure S3. Validation of spatial fidelity and cellular integrity of *L. minor* mother during the pPTFE imprinting using the PNP. The microscope images of a **(A)** fresh mother frond and **(B)** the Hematoxylin and Eosin-stained tissue recovered after imprinting.

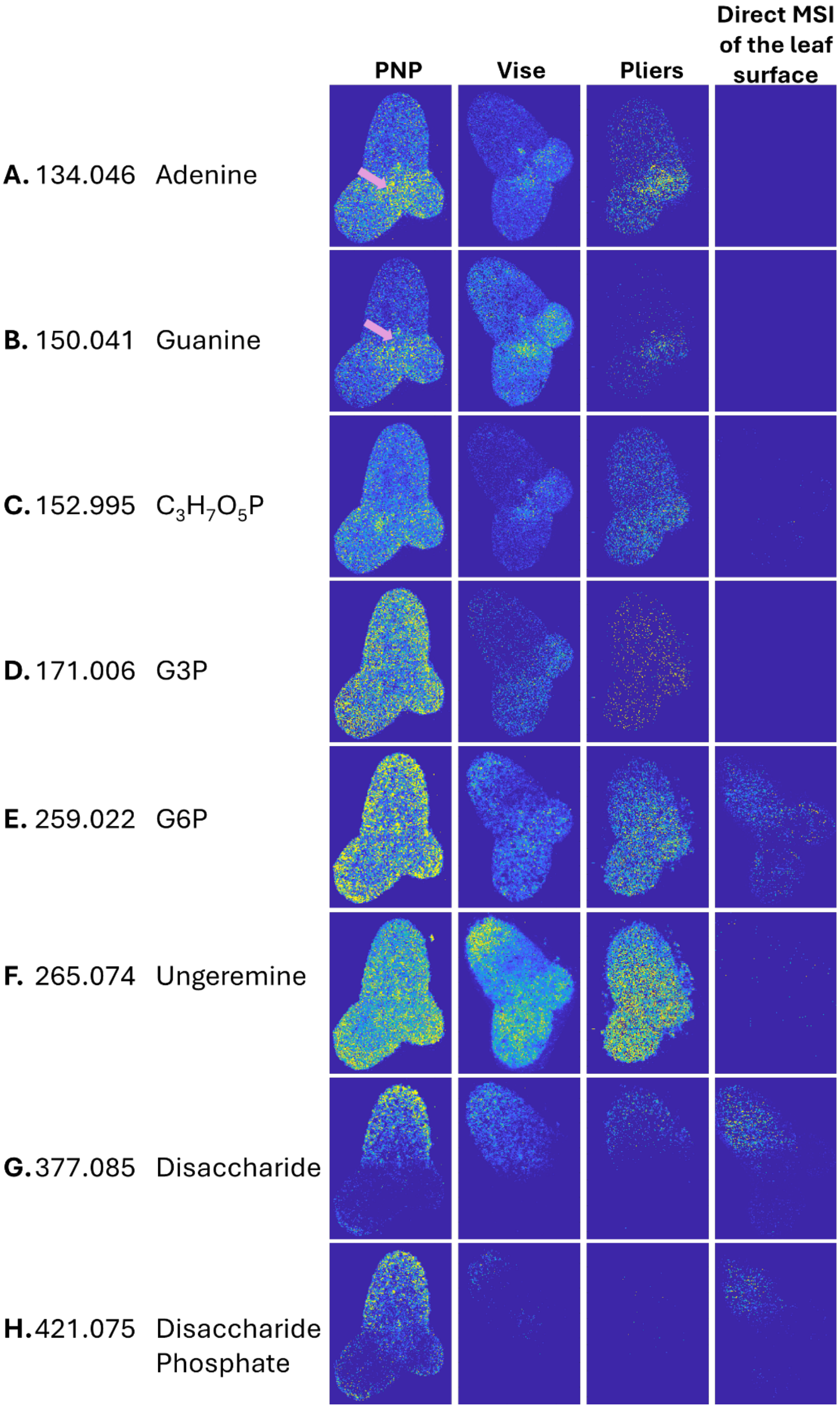

Figure S4. Comparison of tissue-specific localization of selected internal metabolites in *L. minor* using three distinct imprinting techniques alongside direct MSI analysis of the adaxial leaf surface.
